# Motivational dynamics of healthy living: a qualitative study based on self-determination theory

**DOI:** 10.1186/s12889-026-27586-9

**Published:** 2026-04-29

**Authors:** Elif Özer Kenan, Necla Yılmaz

**Affiliations:** 1 Department of Medical Services and Techniques, Refahiye Bahar Yıldırım Vocational School of Health Services, Erzincan Binali Yıldırım University, Erzincan, Türkiye; 2 Department of Healthcare Management, Faculty of Economics and Administrative Sciences, Süleyman Demirel University, Isparta Türkiye

**Keywords:** Healthy living, Motivation, Healthy living motivation, Healthy living behaviours, Self-Determination Theory

## Abstract

**Background:**

Healthy living behaviours play a central role in preventing chronic disease and improving population health; therefore, understanding the quality of motivation behind these behaviours is essential. This study aimed to explore the motivational factors that shape the adoption and maintenance of healthy living behaviours within the framework of Self-Determination Theory.

**Methods:**

A qualitative phenomenological design was employed. Semi-structured interviews were conducted with 18 adults living in different regions of Türkiye who had experience with healthy living. Data were analyzed using thematic analysis.

**Results:**

Motivation for healthy living was found to be multidimensional and emerged from the interplay of intrinsic and extrinsic factors. Participants reported that concerns related to aging and health, physical and psychological well-being, social desirability, family and social support, illness experiences, and cultural and spiritual values influenced their motivation. Some factors reflected intrinsic motivation, whereas several external influences were internalised over time and became more autonomous. Satisfaction of autonomy, competence, and relatedness needs played a central role in the sustained practice of healthy living behaviours.

**Conclusions:**

Motivation for healthy living is shaped not only by individual benefits but also by social, cultural, and spiritual contexts. The findings offer new insight into the process of motivational internalization and may guide public health strategies aimed at strengthening individuals’ motivational resources.

**Trial registration:**

Not applicable. This study was not a clinical trial.

**Supplementary Information:**

The online version contains supplementary material available at 10.1186/s12889-026-27586-9.

## Introduction

Modern societies are experiencing rapid changes in daily living patterns. Although technological conveniences make life easier, they also reduce opportunities for physical activity. At the same time, busy work schedules, stress, and unhealthy eating habits negatively affect Healthy Living (HL). As a result, obesity, diabetes, hypertension, and mental health problems continue to increase worldwide [1, 2]. According to recent reports from the World Health Organization (WHO), nearly one-third of adults do not engage in sufficient physical activity, making physical inactivity one of the leading contributors to the global burden of disease [3]. Hallal et al. [4] also emphasize that physical inactivity has reached alarming levels worldwide.

The literature shows that increased consumption of ultra-processed foods is strongly associated with obesity and metabolic disorders [5]. Findings from Global Burden of Disease studies identify unhealthy diets, physical inactivity, and high stress levels as major risk factors for population health [6]. Rapid changes in modern lifestyles, such as prolonged screen time and constant online engagement, also negatively affect psychological well-being [7]. Taken together, these issues highlight the urgent need to promote Healthy Living Behaviours (HLB) at the population level.

Recent research indicates that individuals do not maintain HLB solely because they possess knowledge or awareness. Instead, the quality of their motivation plays a key role in sustaining these behaviors [8, 9]. Understanding how motivation develops and how it can be maintained over time is therefore essential for designing effective public health strategies. For this reason, focusing on the type of motivation, rather than simply its presence, is important for understanding behaviour change.

Self-Determination Theory (SDT) offers a comprehensive framework for explaining how motivation supports the adoption and maintenance of HLB. SDT proposes that the fulfilment of three basic psychological needs-autonomy, competence, and relatedness-helps individuals develop more internalised, stable, and sustainable forms of motivation [10]. Recent studies show that supporting these needs increases long-term engagement in behaviours such as physical activity, healthy eating, and stress management [8, 11].

In the Turkish context, research on how individuals understand and sustain HLB, what motivates them, and how social and cultural environments shape their motivation remains limited. However, motivation is not solely an individual process. It is also shaped by social norms, family structures, cultural values, and spiritual beliefs. To design effective public health programs, we need to understand which psychological, social, and cultural resources people rely on to maintain health-promoting behaviours. This study aims to address this gap.

Despite growing evidence on the importance of motivation for sustaining HLB, the literature lacks an in-depth qualitative understanding of how different types of motivation are experienced, internalised, and sustained within specific sociocultural contexts. In particular, little is known about how individuals in Türkiye make sense of HL motivation over time and how the basic psychological needs of autonomy, competence, and relatedness interact with social, cultural, and spiritual influences in shaping this process. This gap limits the development of context-sensitive public health interventions grounded in motivational theory. Therefore, a clearer understanding of the motivational dynamics underlying HL is needed.

In this study, we aim to explore the factors that shape individuals’ motivation to adopt and sustain HLB and to examine how the needs for autonomy, competence, and relatedness are experienced in this process. Our findings are expected to contribute to the development of public health programs that are more effective, accessible, and capable of strengthening individuals’ motivational resources.

### Theoretical framework: self-determination theory in understanding healthy living motivation

Healthy living motivation (HLM) refers to individuals’ intrinsic or extrinsic drives to adopt and maintain HLB in order to preserve or improve their physical, mental, and social well-being [12–15]. This motivation is not merely a temporary desire to achieve a specific goal; rather, it represents a deeply integrated and enduring orientation in an individual’s life. Behaviours such as engaging in regular exercise, maintaining a balanced diet, obtaining sufficient sleep, and coping with stress in healthy ways are more easily initiated and sustained over the long term when adequate motivation is present [16–18]. Research demonstrates that motivation plays a critical role in predicting, understanding, and modifying HLB [19, 20].

The theoretical foundation of this research is grounded in SDT, a comprehensive theory that conceptualizes motivation in terms of different qualities and levels. SDT emphasizes that motivation is one of the most important factors determining the persistence and intensity of human behaviour [21]. Within this framework, motivation is broadly classified as intrinsic or extrinsic [10, 22]. Intrinsic motivation refers to engaging in an activity for its inherent satisfaction rather than for external rewards or pressures. While performing the activity, individuals experience curiosity, interest, enjoyment, or a sense of personal fulfilment. For example, a person who exercises regularly because they genuinely enjoy it or feel good while doing it demonstrates intrinsic motivation.

Extrinsic motivation, in contrast, refers to behaviour drives by external factors related to the outcomes of the activity. In such cases, motives such as gaining rewards or avoiding punishment predominate. For instance, an individual who eats healthily primarily because it is recommended by a physician, or who exercises to avoid criticism about their weight, is mainly guided by extrinsic motivation.

A distinctive feature of SDT is its assertion that extrinsic motivation can be internalised to varying degrees. Beginning with external regulation and progressing through introjected and identified regulation, the process may ultimately reach integrated regulation, at which point motivation acquires a level of autonomy comparable to intrinsic motivation. During this process of internalization, a behaviour that is initially undertaken for external reasons becomes more stable and enduring as it aligns with the individual’s values and identity. Autonomous forms of motivation are therefore critically important for the sustainability of HLB [23].

SDT also emphasizes three basic psychological needs that support motivation [24]:

- Autonomy refers to the need to choose and regulate one’s own behaviours. For example, following an exercise program that one has personally selected supports this need.
- Competence refers to the need to feel effective and capable. Achieving goals related to HLB or observing physical improvement reinforces this sense of competence.
- Relatedness refers to the need to establish meaningful connections with others and to receive social support. Family encouragement or exercising with friends can help fulfil this need.

In summary, when these needs are satisfied, individuals are more likely to adopt and self-regulate HLB, which in turn helps them sustain these behaviours over the long term and achieve positive outcomes. In the context of HL, the theory suggests that when individuals experience freedom of choice regarding HLB, feel competent in performing them, and experience a sense of belonging during the process, the likelihood of maintaining HL over time increases [24, 25]. Therefore, fulfilling the basic psychological needs of autonomy, competence, and relatedness is considered essential for strengthening individuals’ intrinsic motivation.

**Fig. 1:**
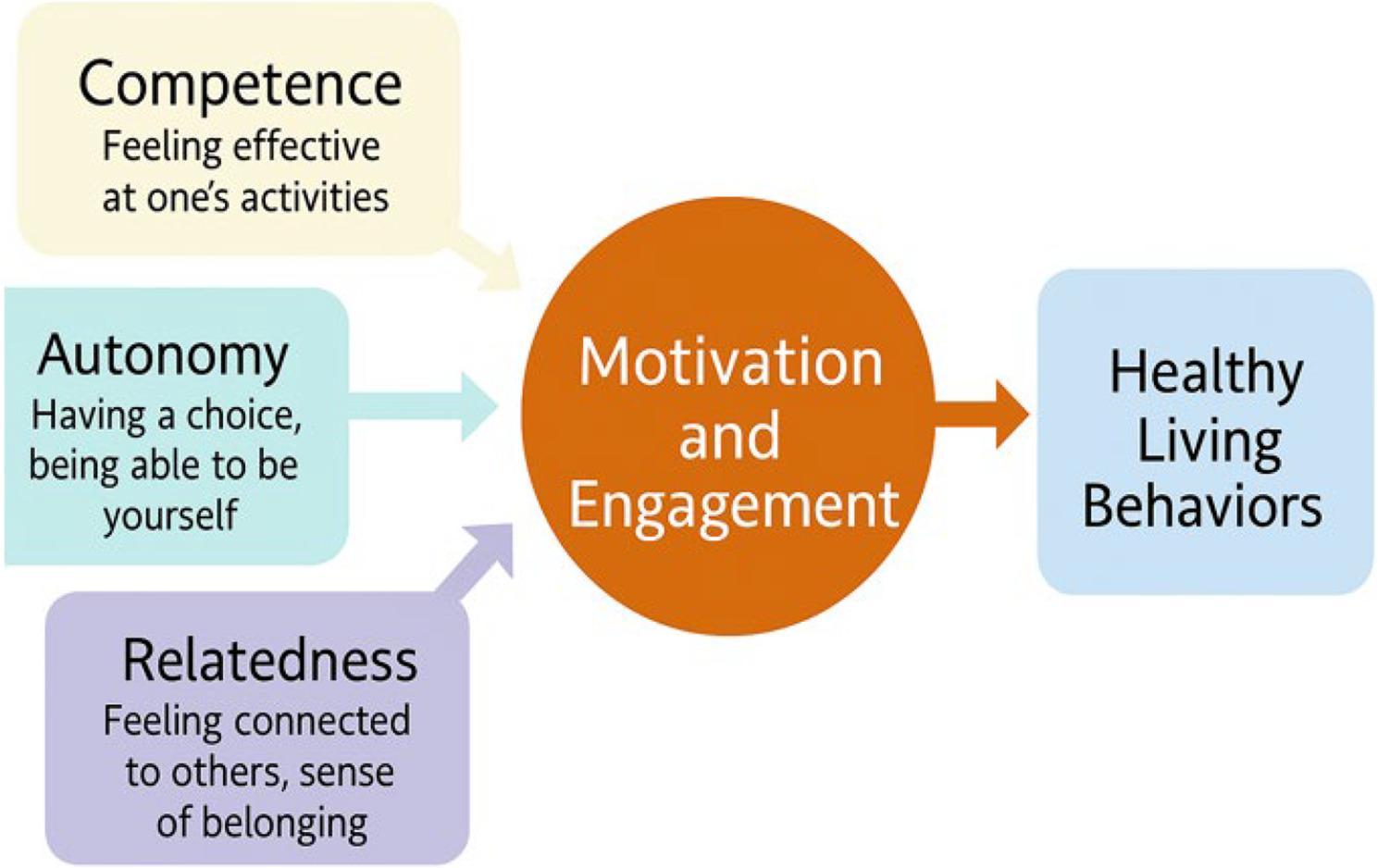
Relationship between basic psychological needs, motivation, and HLB within the framework of SDT. Source: Author own work

Figure 1 conceptually illustrates how the needs for autonomy, competence, and relatedness, based on SDT, support HLB through motivation and engagement.

Figure 1 conceptually illustrates how the basic psychological needs of autonomy, competence, and relatedness support motivation for HL and how this motivation, in turn, fosters individuals’ cognitive, emotional, and behavioural engagement in HLB.

## Method

### Research design

This study used a qualitative research design to gain a deeper understanding of the factors that motivate individuals to pursue HL. A qualitative approach was chosen because it allows individuals to express their experiences, perceptions, and motivational processes from their own perspectives. The research adopted a phenomenological approach to explore the essence of participants’ experiences related to motivation for HL.

Consistent with this aim, the study is grounded in an interpretivist epistemological perspective, which assumes that individuals construct meaning through their lived experiences and social contexts. Accordingly, the research does not seek to identify a single objective reality; rather, it aims to understand how participants interpret and make sense of their motivation for HL [26, 27]. Within this framework, participants’ experiences were explored and interpreted using the conceptual lens provided by SDT.

### Participants

The study group consisted of 18 adult individuals residing in different provinces of Türkiye. The sampling strategy was designed in line with the phenomenological nature of the study and was based on purposive sampling. Specific criteria were established to recruit individuals with sufficient knowledge and experience related to the research topic [28, 29]. These criteria included having experience related to HL (engaging in HLB for at least three years, participating in physical activity at least three days per week, and consuming at least three meals per day), being over 18 years of age, and volunteering to participate in the interview. Exclusion criteria included severe communication difficulties, cognitive impairments, and health conditions unrelated to the focus of the study.

Participants were invited to participate in the study through social media announcements, health-related communities, and the researcher’s personal network. To ensure diversity, we aimed to include individuals with different ages, genders, occupations, and lifestyles. Patton [30] states that a sample size can be considered sufficient when the data obtained from participants begin to repeat. Accordingly, data saturation was used as the criterion to determine the number of participants in this study. The data began to repeat by the 16th interview; therefore, the final sample consisted of 18 participants. Participants were assigned alphanumeric codes (K1, K2, …) to ensure anonymity.

### Data collection process

Data collection was carried out between May and July 2024. All interviews were conducted by the primary researcher. The researcher had prior academic experience related to HL awareness and was knowledgeable about the topic. However, there was no personal or professional relationship with the participants before the interviews. This helped minimize potential power imbalances and allowed participants to share their experiences more openly.

Data were collected through semi-structured interviews, which are commonly used in phenomenological research [31]. Open-ended questions were developed based on a review of the literature and the three basic psychological needs described in SDT. However, questions were designed to remain open-ended and exploratory in order to capture participants lived experiences. The interview guide used in this study was developed by the research team specifically for this project and refined with expert feedback. Pilot interviews were conducted, and the questions were finalized accordingly. A brief questionnaire was also included to collect demographic information.

Before each interview, participants were informed about the purpose of the study and assured that their personal information would remain confidential. Interviews were scheduled at mutually convenient times. With participants’ consent, an audio recorder was used to prevent data loss and enhance the credibility of the data [32]. In total, 726 min and 4 s of audio recordings were obtained. These recordings were transcribed digitally, resulting in a dataset of 155 pages. The transcripts were read several times to ensure data accuracy before the coding process began.

All interviews were conducted in Turkish. Participant quotations presented in the manuscript were translated into English by the research team for reporting purposes. Care was taken to preserve the original meaning and tone of the participants’ expressions during the translation process.

### Data analysis

The data were analysed using the six-phase thematic analysis approach proposed by Braun and Clarke [33]. The analysis followed a hybrid strategy that combined inductive coding with theoretically informed interpretation.

In the first phase, all interviews were transcribed and read several times to ensure familiarity with the data. In the second phase, initial codes were generated inductively from participants’ narratives, allowing patterns and meanings to emerge directly from the data. No predefined coding framework was applied at this stage. In the third phase, similar codes were grouped to form preliminary themes. In the fourth phase, these themes were reviewed and refined through comparison with the entire dataset. During this stage, the emerging themes were interpreted in relation to the conceptual framework of SDT to ensure theoretical coherence.

After the initial themes were developed, a second round of coding was conducted. This step aimed to enhance analytic consistency and to verify the alignment between the coded extracts and the thematic structure. The second coding process corresponded to the review and refinement stages of the thematic analysis framework and contributed to strengthening the credibility of the analysis.

In the final phases, themes were defined, named, and supported with direct participant quotations. The coding structure and themes were reviewed by four faculty members, and the final themes were agreed upon through researcher consensus. During analysis, theoretical constructs were used as an interpretive framework for understanding emergent themes. This approach ensured a coherent link between theory, data collection, and findings.

### Trustworthiness and rigor

Methodological rigor was evaluated according to the trustworthiness criteria proposed by Lincoln and Guba [34], including credibility, transferability, dependability, and confirmability. Credibility was supported through prolonged engagement with the data, repeated readings of transcripts, and iterative coding procedures. A second round of coding was conducted to enhance analytic consistency. The coding structure and thematic organization were reviewed by four faculty members, and the final themes were established through researcher consensus.

Transferability was addressed by providing detailed descriptions of the research context, participant characteristics, and analytic procedures, enabling readers to assess the applicability of the findings to other contexts. Dependability was strengthened through transparent documentation of each stage of the research process, including sampling decisions, interview procedures, and analytic steps. Confirmability was supported by grounding interpretations in direct participant quotations and maintaining clear alignment between the raw data and the thematic conclusions. Reflexivity was also considered throughout the research process. The primary researcher acknowledged prior academic engagement with HL research and remained attentive to potential assumptions during data collection and analysis.

## Results

The sample consisted of 18 participants, of whom 10 were male and 8 were female. Participants’ age, occupation, and HLS characteristics are presented in Table 1. When occupations were examined, the largest group consisted of participants working in academia. In addition, individuals working in the private sector, healthcare, public service, and skilled/manual labour were also included in the study. The duration of maintaining a healthy lifestyle ranged from 3 to 25 years. Participants reported engaging in physical activity between 3 and 7 days per week, with most indicating regular physical activity on 3–4 days per week. Regarding eating habits, most participants reported consuming three main meals and one to two snacks per day.

**Table 1:** Demographics and HLB of the Participants

Participant Code	Occupation	Adoption Period of Healthy Living (years)	Physical Activity (Per Week)	Meals + Snacks
K1	Private Sector	25	6	2 + 1
K2	Academia	5	3	3
K3	Healthcare Support Services	3	4	3 + 2
K4	Academia	3	3	3 + 1
K5	Healthcare Professional	6	4	2 + 1
K6	Not Currently Employed	3	6	3 + 1
K7	Academia	4	4	3 + 1
K8	Worker	20	4	2 + 2
K9	Academia	3	3	2 + 1
K10	Academia	3	4	3
K11	Academia	10	5	3
K12	Healthcare Professional	20	4	2 + 1
K13	Academia	3	4	2 + 1
K14	Public Service	8	5	2 + 1
K15	Academia	3	3	2 + 1
K16	Public Service	3	7	3
K17	Public Service	10	6	2 + 1
K18	Public Service	5	6	3 + 1

### Thematic results

**Fig. 2:**
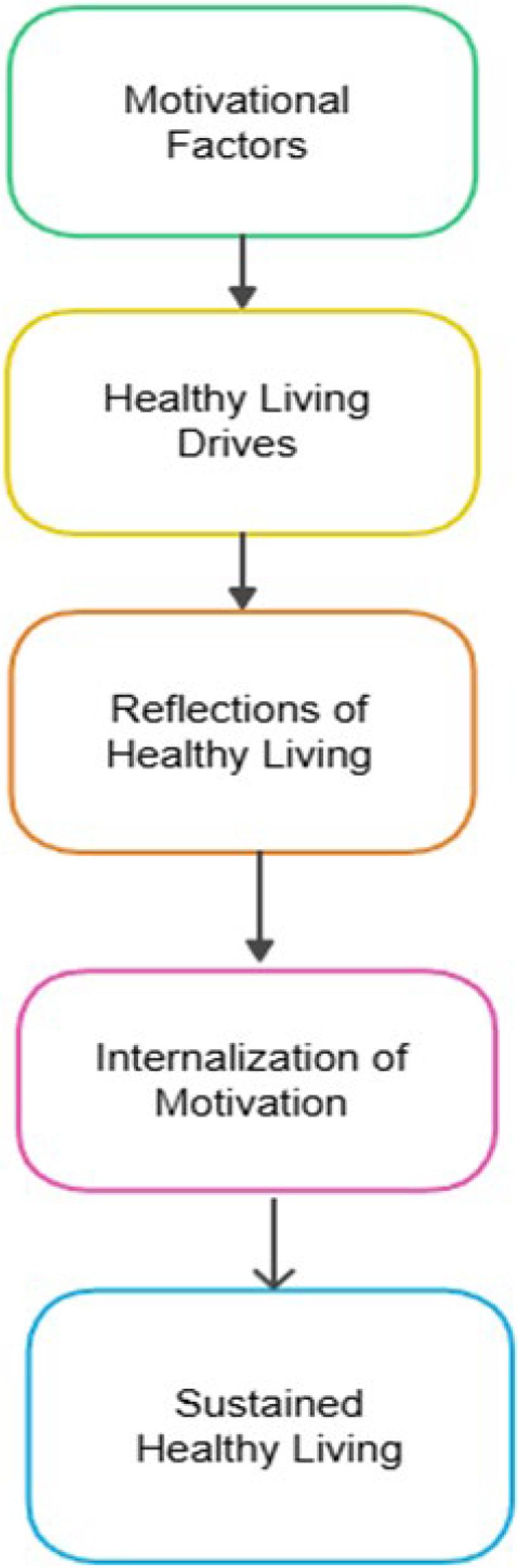
Process model of healthy living motivation. Source: Author own work

As a result of the data analysis, three main themes related to the process of making sense of healthy living emerged: factors motivating healthy living, healthy living drives, and reflections of healthy living. The relationships among these themes are illustrated in Fig. 2. The figure presents the motivational process underlying HLB. Motivational factors shape individuals’ initial orientation toward healthy living, while various experiences and turning points strengthen this motivation. As individuals continue these behaviours, they reflect on the physical, psychological, and social benefits they experience. Over time, these experiences contribute to the internalization of motivation, which supports the sustained maintenance of HLB.

### Theme 1. Factors motivating healthy living

**Fig. 3:**
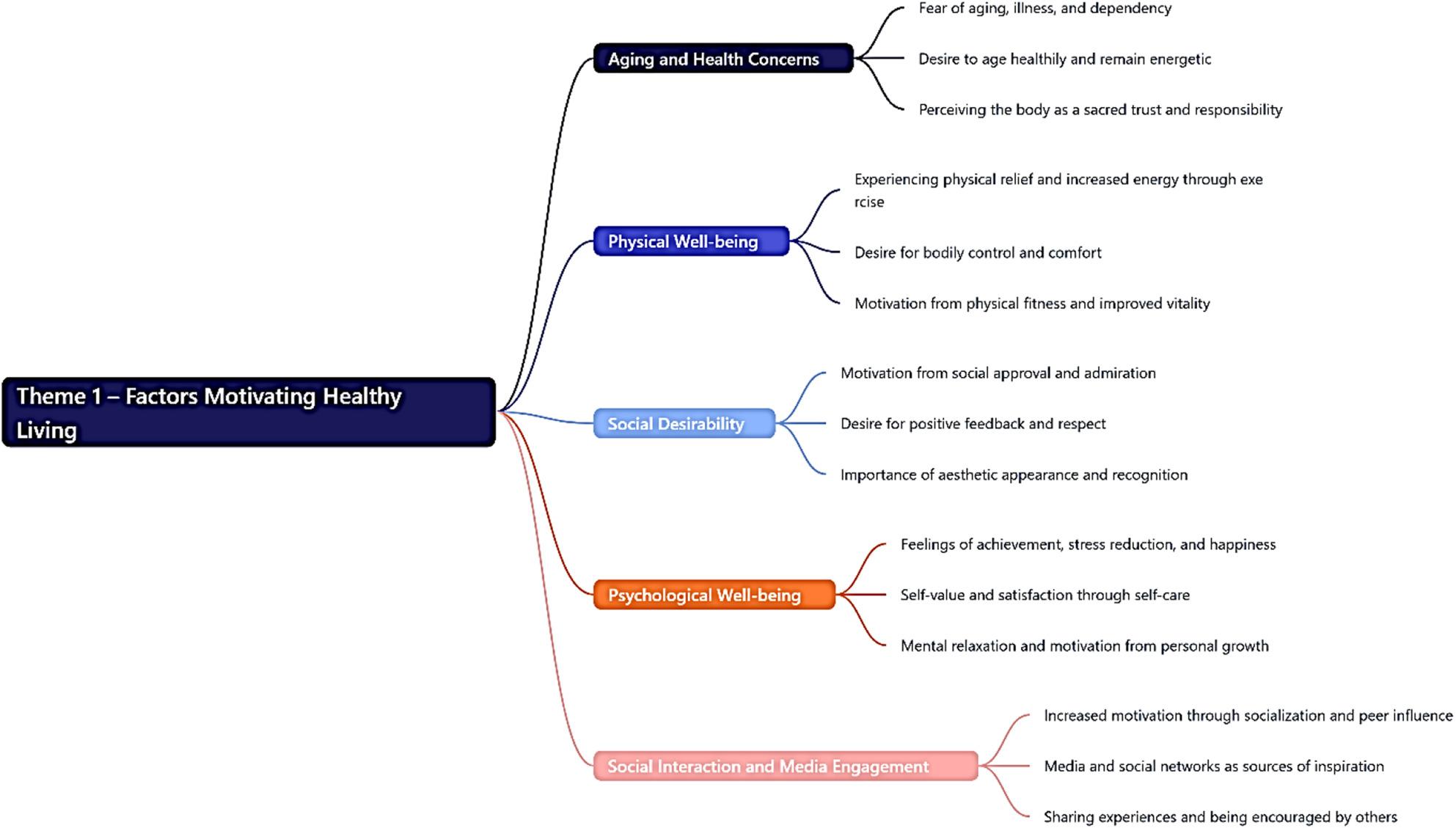
Coding tree for theme 1: factors motivating healthy living. Source: Author own work

Figure 3 illustrates the theme, subthemes, and codes derived from participants’ expressions regarding their motivation to adopt and maintain HL. Participants’ narratives indicated that motivations for adopting HL are multifaceted. Concerns about ageing, illness, and becoming dependent on others emerged as significant drivers of engagement in HLB. One participant expressed this concern by stating, *“I don’t want to be dependent on my children. They shouldn’t have to take care of me; I can’t take away their time.”* (K1). Similarly, perceiving the body as a trust to be preserved was another important motivator: *“I use my body with the motivation of not betraying the entrusted body given to me.”* (K11). Physical well-being was also frequently emphasized as a source of motivation. Participants associated regular exercise and balanced nutrition with physical comfort, increased energy, and greater ease of movement. One participant explained, *“On the days I don’t exercise*,* I generally feel worse. Physically*,* I feel much better when I do.”* (K4). Others highlighted the perceived benefits of HL for sexual health, stating, *“Exercise and nutrition increase testosterone*,* and this has a positive effect on my sexual life.”* (K14).

Social approval and positive feedback were important motivators for some participants. The desire to be appreciated, accepted, and respected by others reinforced their commitment to HL. For instance, one participant noted, *“When people say things like ‘You look great*,* you’ve lost weight*,*’ of course I enjoy that social acceptance.”* (K13). The belief that maintaining a healthy body enhances one’s social value was also evident: *“When I take good care of my body I feel like a valuable and desirable person.”* (K2). Psychological well-being also played a central role in motivating HLB. Participants frequently described experiencing a sense of achievement, reduced stress, and emotional relief through physical activity. For example, one participant stated, *“The best thing life has brought me is exercise… even spending twenty minutes on a mat at home makes me feel truly valuable.”* (K3). Others emphasized goal setting and personal development: *“There’s always a new goal in sports*,* and achieving those goals brings immense satisfaction.”* (K14).

Finally, social interaction and media influence were identified as meaningful motivators. Many participants reported that engaging in activities such as sports expanded their social networks and created opportunities for connection and personal growth. As one participant noted, *“Through sports*,* my social circle grows; connecting with different people enriches me.”* (K11). Inspirational media content also contributed to initiating motivation: *“When Tülin Şahin said she was 45*,* I thought*,* I wish the gym were open-I’d go too.”* (K4). Additionally, social media enabled reciprocal motivation among peers: *“When I share my experiences*,* my friends get motivated-and they also motivate me.”* (K5). Overall, the findings indicate that the needs for autonomy, competence, and relatedness play a central role in motivating HLB.

### Theme 2. Healthy living drives

**Fig. 4:**
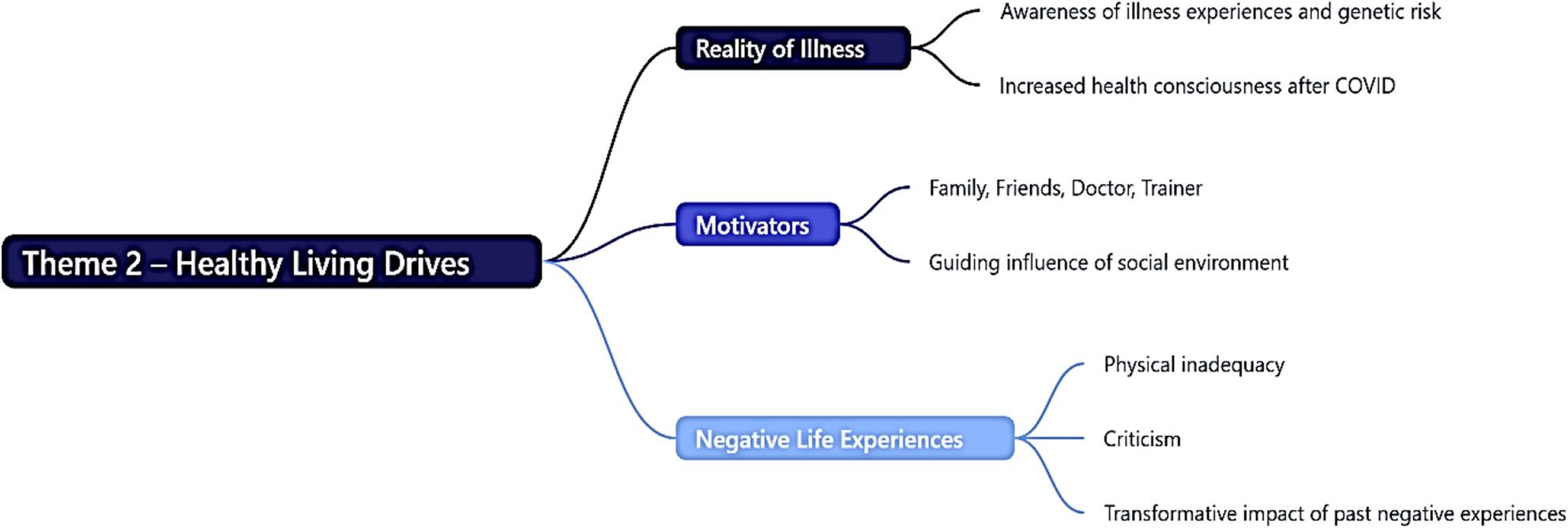
Coding Tree for theme 2: healthy living drives. Source: Author own work

Figure 4 presents the theme, subthemes, and codes that describe what initially encouraged or strengthened participants’ commitment to HL. The first subtheme, reality of illness, shows that past health problems, physical discomfort, and awareness of genetic risks served as important turning points for many participants. Some participants described personal health concerns, such as *“My insulin levels came out high. Just feeling that my health was at risk was enough for me.”* (K7) and *“I approach things cautiously because I’m aware of my genetic predispositions.”* (K13).

The second subtheme, motivators, emphasizes the role of social support. Family members, friends, physicians, and trainers were mentioned as key sources of encouragement. Their support helped participants develop healthier habits and maintain consistency in HLB. For example, one participant stated, *“My doctor really motivated me. If it had been someone else*,* I wouldn’t have been able to adapt this well.”* (K12), while another noted, *“If you’re going to exercise*,* you should do it with someone who knows what they’re doing.”* (K8). Some participants were also influenced by observing the limitations experienced by others. As one participant explained, after observing the condition of older individuals, *“I did not want to become like them in the future.”* (K5).

The third subtheme, negative life experiences, highlights how past challenges and social pressures encouraged participants to make changes. Some participants described feeling physically limited or receiving negative comments about their appearance, which motivated them to improve their health. For example, one participant noted, *“While tying my shoes*,* I felt like a 70-year-old… facing that reality pushed me to take action.”* (K10), and another shared, *“When my uncle said*,* ‘Did you gain weight?’*,* that was the last straw.”* (K6). Overall, this theme indicates that HLM is often shaped by personal health experiences, support from others, and challenging life events that trigger the desire for change.

### Theme 3. Reflections of healthy living

**Fig. 5:**
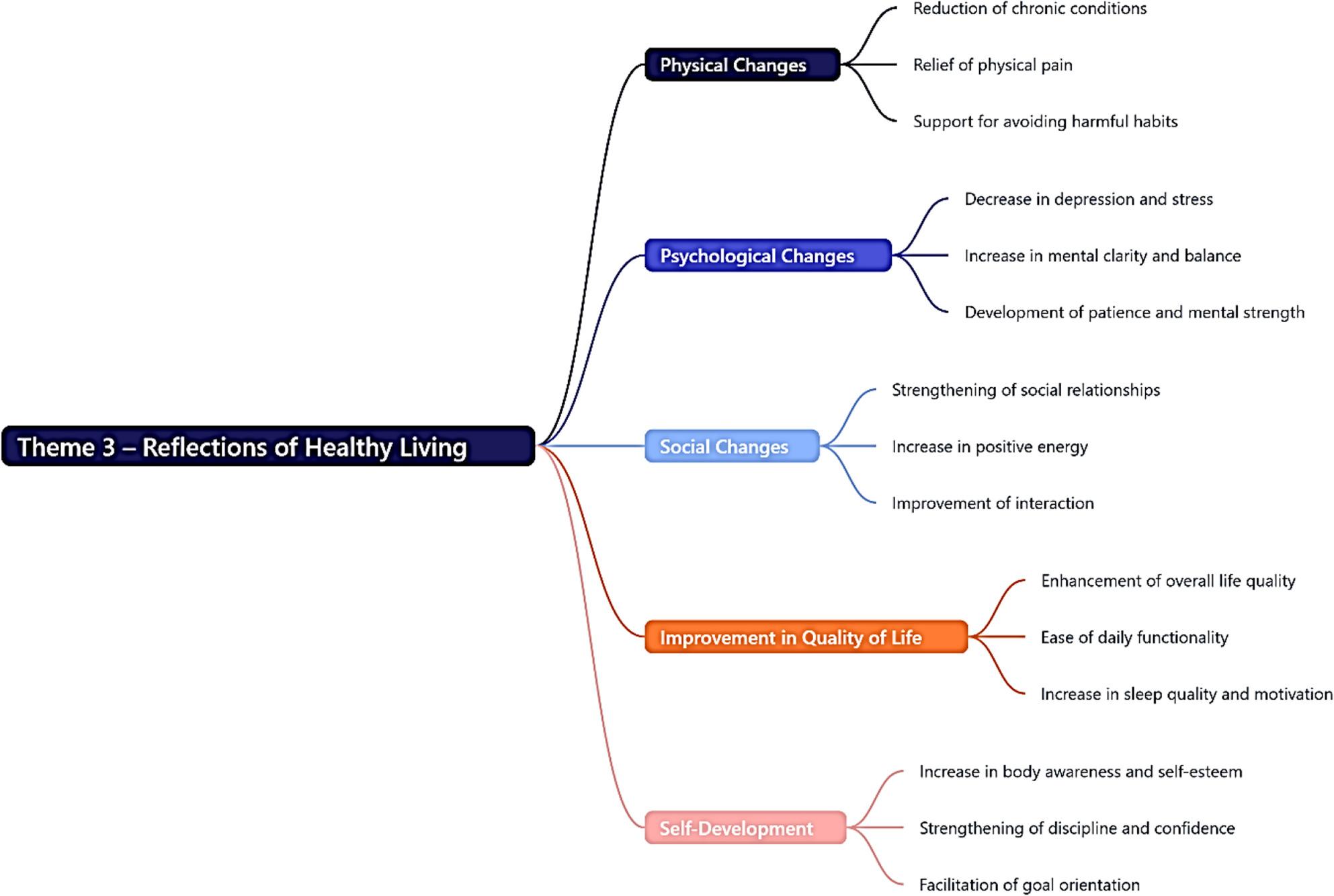
Coding tree for theme 3: reflections of healthy living. Source: Author own work

Figure 5 illustrates the theme, subthemes, and codes based on participants’ reflections on how HL has affected their lives. These reflections are grouped into five subthemes: physical changes, psychological changes, social changes, improvement in quality of life, and self-development.

The physical changes subtheme highlights that regular physical activity, and healthy habits contribute to reduced pain and improved physical health. Many participants reported feeling physically better and experiencing illness less frequently. For example, *“When I don’t do yoga*,* I get pain in my back*,* waist*,* and muscles… If I skip exercise*,* the pain returns immediately.”* (K5). Similarly, one participant explained, *“After I started exercising*,* I began getting sick less often compared to people around me.”* (K9). Participants also referred to dietary habits as part of these physical improvements, emphasizing balanced meals, reducing processed foods, and paying attention to portion sizes. Some described how healthier eating patterns improved their energy levels and daily functioning. As one participant expressed, *“When I started paying attention to what I eat*,* I realized how much it affects my daily energy. Eating balanced meals makes me feel lighter and more active during the day.”* (K8). These accounts suggest that participants perceived both physical activity and nutrition as contributing to improved physical well-being.

The psychological changes subtheme indicates that HL had a strong positive influence on participants’ mood and mental clarity. Many described feeling less stressed, more focused, and emotionally balanced. As one participant stated, *“It’s like a natural medicine… I both clear my mind and do something for my health. I’ve made my toughest decisions while walking.”* (K15). Another participant noted, *“You become more patient*,* calmer. You start looking for solutions to negativity.”* (K9). In the social changes’ subtheme, participants explained that maintaining HL improved their relationships with others. They reported feeling more confident and open, which contributed to better communication and stronger social connections. One participant shared, *“As you meet more people and share things together*,* your self-confidence and motivation increase. You build stronger and more genuine relationships.”* (K18). Another participant emphasized the social impact of positive energy, stating, *“Because you are happy*,* you give positive energy to others. For example*,* when a friend comes to you feeling cheerful and energetic*,* you would prefer to spend time with them rather than someone who constantly complains and feels unwell”* (K17).

The improvement in quality-of-life subtheme reflects how healthy habits made everyday life easier and more enjoyable. Participants frequently mentioned better sleep, higher energy levels, and a stronger sense of motivation. For instance, one participant stated, *“Your quality-of-life increases… Once you reach that standard*,* you make an effort not to let it go.”* (K15). Another participant added, *“Exercise gives me joy*,* energy… I don’t get tired I honestly don’t.”* (K16).

Finally, the self-development subtheme reveals that participants developed a stronger sense of self through HL. They described increased self-confidence, greater self-discipline, and enhanced body awareness. One participant expressed, *“Realizing that you’re the one in control of your own body is an incredible thing.”* (K2). Another highlighted the importance of discipline: *“Discipline in nutrition… It’s one of the hardest things I’ve ever faced. I think there’s very little that someone who can manage that can’t achieve.”* (K18). In summary, participants described how maintaining HL brought meaningful changes not only to their physical health but also to their psychological well-being, social relationships, and self-perception. These reflections suggest that the benefits of HL extend beyond physical health and contribute to a stronger, more balanced, and more confident sense of self.

## Discussion

In this study, the multidimensional nature of HLM was examined qualitatively, and a range of motivational factors were identified under three main themes based on participants’ experiences. The findings indicate that the processes of adopting and maintaining HL are shaped by the interaction of both intrinsic and extrinsic factors. As illustrated in the thematic process model (Fig. 2), motivation for healthy living appears to develop through an interconnected process involving motivational factors, triggering experiences, and the perceived outcomes of healthy living, which together contribute to the internalization of healthy living behaviours. When interpreted within the framework of SDT, these themes provide valuable insights into the continuity and quality of motivation. These findings are also relevant for public health, as they illustrate how individual motivational processes are connected to improvements in population health, the reduction of chronic diseases, and the design of effective health behaviour interventions.

To begin with, some of the motivational elements identified in our study clearly reflect characteristics of intrinsic motivation. For example, participants’ enjoyment of HL, their pleasure in engaging in HL-related activities, and their desire to maintain these behaviours for their own well-being are directly related to intrinsic motivation. Such motivations, which arise from satisfaction with the behaviour itself, are considered the most autonomous and sustainable forms of motivation. Ryan and Deci [22] emphasize that individuals’ sense of responsibility and self-worth strengthens intrinsic motivation and supports the long-term maintenance of behaviours. Similarly, in our findings, internal sources such as self-care and the experience of feeling happy emerged as important factors that increased participants’ motivation.

The literature also indicates that HL contributes to greater happiness and life satisfaction, and that the desire to maintain this sense of well-being can act as a powerful motivator [35]. Most participants in our study supported this perspective by reporting that they experienced psychological well-being associated with HL, which increased their commitment to maintaining this lifestyle. This finding is also important from a public health perspective because intrinsic motivation enables individuals to sustain behaviours without relying on external rewards, providing an important foundation for lower-cost and more sustainable public health interventions.

On the other hand, some of the motivational factors identified in this study can be classified as extrinsic motivation, although they operate at different levels of internalization. For example, motivations such as the desire to be liked or the pursuit of respectability primarily reflect outcome-oriented and externally regulated forms of motivation. Similarly, the desire for social approval or the wish to be appreciated by others reflects a tendency to maintain behaviour in response to external rewards [36]. Although these drivers are essentially extrinsic, under certain conditions they may evolve into introjected or identified forms of motivation.

Participants’ accounts suggest that compliments and approval from the social environment initially functioned as external motivators but gradually contributed to their sense of self-worth and self-esteem, thereby gaining more internal significance. For instance, one participant initially aimed to lose weight “so that others would like her,” but later reported, *“being healthy makes me feel better*,*”* illustrating how extrinsic motivation can become internalised. Deci and Ryan [21] note that social praise and recognition are important external factors that can enhance motivation, but they may eventually become integrated into an individual’s value system.

Consistent with this perspective, our findings indicate that although the need for social approval initially encouraged participants to adopt HLB, these behaviours gradually acquired personal meaning as participants experienced their benefits, ultimately becoming a source of intrinsic satisfaction. Furthermore, Ryan and Deci [22] emphasize that the need for social acceptance plays a significant role in the internalization of goals such as HL. Participants in our study similarly reported that positive feedback from their social environment not only satisfied their need for recognition but also helped them integrate HL habits into their personal identity.

These findings suggest that when extrinsic motivation is successfully internalised, an initially external reward can evolve into genuine self-motivation. This process also helps explain why external incentives, such as short-term campaigns, often fail to produce lasting effects in community-wide behaviour change programs, and why aligning interventions with individuals’ personal values is essential.

The findings of this study demonstrate that motivation for HL is closely associated with the three basic psychological needs emphasized by SDT. In participants’ narratives, the need for autonomy was particularly salient. The desire to avoid dependency on others in older age and to maintain control over one’s own health emerged as key drivers in sustaining HLB. Having the freedom to make one’s own behavioural choices is a critical factor that supports motivation, and when this need is satisfied, motivation becomes more internalised and sustainable [37]. This finding is consistent with the literature highlighting the positive effects of autonomy support on HLB. For example, a meta-analysis of SDT-based interventions reported that approaches supporting individuals’ sense of autonomy produced significant positive effects on HLB, and that increases in autonomous motivation-rather than controlling forms of motivation-better explained behaviour change [8, 38]. From a public health perspective, using autonomy-supportive communication approaches in primary care, community health centres, and school-based programs may therefore represent an important strategy for strengthening the sustainability of HLB.

The study also indicates that the need for competence plays a central role in motivation. Participants reported that the sense of achievement gained from reaching goals through regular exercise and healthy eating, as well as observing physical improvements, increased their motivation. This reflects the fundamental human need to feel effective and capable. The literature consistently shows that satisfaction of the need for competence enhances motivation, particularly in the context of exercise participation. For example, Teixeira et al.’s [9] comprehensive review reported that greater satisfaction of competence and higher intrinsic motivation related to physical activity were positively associated with exercise adherence. In this study, participants’ statements such as *“there’s always a new goal*,* and the satisfaction of achieving them is immense”* clearly illustrate how fulfilling the need for achievement contributes to motivation. This finding supports theoretical arguments suggesting that satisfying the need for competence generates higher-quality psychological energy, thereby increasing the likelihood of sustaining behaviour [23].

Several participants reported that social support and interaction played a key role in maintaining HL. For example, engaging in physical activity with family members or friends was described as a factor that helped sustain motivation, while receiving appreciation and admiration from others contributed to feelings of being valued. This reflects the third basic psychological need described in SDT-relatedness. According to SDT, a sense of belonging and the development of social connections foster more intrinsic motivation and support the continuity of behaviour [39]. Similarly, previous studies indicate that social connectedness and social support encourage both participation in and maintenance of physical activity [40]. In our study, social support elements-particularly family members, friends, and professional guidance (e.g., from trainers and physicians)-played an important role in helping participants sustain their HL habits.

Another noteworthy finding concerns the role of negative emotions and experiences in shaping motivation. Our results suggest that negative stimuli, such as fear of death, experiences of illness, or social bullying, can function as powerful triggers for behaviour change. This finding aligns with several theoretical perspectives in health psychology. Terror Management Theory [41] proposes that awareness of mortality can motivate individuals to engage in self-protective behaviours. In other words, individuals may adopt healthier behaviours to distance themselves from thoughts of death. Some participants in our study expressed similar concerns, using phrases such as *“I turn to HL to manage thoughts of death*,*”* which aligns with this theoretical perspective.

Similarly, participants who had experienced serious illness or health-related problems often reported making decisive changes, expressing sentiments such as *“I have to take care of my health now.”* This finding is also consistent with the Health Belief Model, particularly the concepts of perceived severity and perceived threat [41]. According to this model, when individuals perceive a disease as serious and personally threatening, they are more likely to adopt preventive behaviours, such as initiating dietary changes or beginning an exercise routine. Our findings support this perspective, indicating that the perceived threat or experience of serious illness can significantly strengthen HLM.

One noteworthy finding is the influence of cultural and spiritual values on motivation. In particular, the theme we defined as the “entrusted body perception”-the belief that the body is a trust from God-led individuals with strong religious and moral convictions to perceive HL as a duty. This finding can be linked to concepts discussed in the literature on religious coping and spirituality. Researchers such as Pargament [42] and Koenig [43] have noted that religious individuals often view caring for their bodies as a spiritual responsibility, which may positively influence their HLB. Among our participants, those who embraced this perception expressed that maintaining HL was not only a personal obligation but also a commitment to their faith, providing them with an additional source of HLM.

This finding suggests that motivation for HL is not explained solely by physical or psychological benefits; it may also include moral and spiritual dimensions. From an SDT perspective, this form of motivation aligns with identified regulation, in which behaviour is pursued because it is personally meaningful and consistent with one’s deeply held values and beliefs. This type of motivation is considered highly autonomous and contributes significantly to behavioural persistence. Therefore, in cultural contexts where such values are prominent, the influence of cultural and spiritual dimensions on motivation should not be overlooked. These findings also indicate that public health programs should incorporate cultural sensitivity. In culturally diverse societies such as Türkiye, communication strategies that align with religious and cultural values may enhance the effectiveness of behaviour change interventions.

Another important finding concerns the influence of contemporary factors, particularly social media, on HLM. Although traditional motivational theories do not explicitly address digital environments, social media clearly shapes individuals’ health perceptions and behaviours. A recent study examining the promotion of HLB through social media reported that online social interaction and a sense of community can satisfy individuals’ basic psychological needs and increase participation in health-promoting behaviours [44]. Some participants in our study reported that content encountered on platforms such as Instagram and YouTube occasionally inspired them to take action. This observation is significant from a health communication and public health perspective, as it suggests that individuals can be motivated through carefully designed messages. However, poorly designed or overly idealized content may produce the opposite effect. While social media was generally perceived as motivating by participants in this study, this area remains open for further research.

Our findings are largely consistent with previous studies in the literature, while also offering several novel contributions. For example, extrinsic sources of motivation such as appearance-related concerns and the need for social approval are frequently observed in motivations related to exercise and diet, as reported in earlier research [45, 46]. However, our study not only confirms the presence of these motivations but also illustrates how they evolve throughout the behavioural process. Some participants initially adopted HLB primarily to lose weight or to achieve a more aesthetic appearance. Over time, however, their experiences led them to recognize the intrinsic value of health itself, resulting in a transformation in the nature of their HLM. This transformation illustrates the process of internalization-a key concept in motivation research-through real-life examples. Such dynamic shifts in motivational orientation remain relatively underexplored in the literature, and our findings contribute to this emerging perspective by highlighting the fluid nature of motivation over time.

When interpreted through the lens of SDT, the findings further support the central role of autonomous motivation in sustaining HLB. Participants’ narratives suggest that the most durable behavioural changes were typically drives by motivations grounded in personal meaning and enjoyment. For example, an individual who begins a diet solely because of medical advice may discontinue it once physician supervision ends. However, if that individual begins to experience increased energy and emotional well-being because of the diet, these intrinsic rewards may help sustain the behaviour. Similarly, in our study, regardless of participants’ initial motivations, internal satisfaction emerged as a key factor influencing their decision to maintain healthy behaviours. This observation aligns with Ryan and Deci’s [22] argument that “healthy living enhances one’s sense of accomplishment, thereby serving as a continual source of motivation.” One participant who experienced positive bodily changes through exercise described this self-sustaining motivational cycle by stating, *“As I notice the difference*,* I feel happier and more motivated.”*

Considering that interventions based on social media are increasingly used in public health, this finding may also provide a foundation for policies aimed at promoting digital health literacy and supporting the development of motivational health communication content.

### Strengths and limitations

This study offers several strengths that contribute to understanding the motivational dynamics underlying HLB. A key strength is the use of a phenomenological qualitative design, which allowed participants to articulate their lived experiences in depth and enabled the exploration of nuanced motivational processes that are often overlooked in quantitative research. The use of SDT as an analytical framework provides a robust theoretical lens through which the interplay between intrinsic and extrinsic motivation can be interpreted, offering a comprehensive understanding of how health behaviours are internalised and sustained. In addition, the diversity of participants in terms of age, occupation, and duration of engagement in HLB enhanced the richness and variability of the data.

However, several limitations should be acknowledged. First, although the sample size was adequate for phenomenological inquiry, it limits the transferability of the findings to broader populations. The sample consisted largely of individuals with higher educational backgrounds and established engagement in HL practices, which may restrict the applicability of the results to individuals with lower health literacy, limited resources, or different sociocultural contexts. Second, the data were self-reported, and participants may have provided socially desirable responses, particularly given the positive social value associated with HL. Third, the study was conducted within a single cultural context, which may shape motivational dynamics in ways that are not generalizable to other cultural settings. Finally, as with all qualitative studies, the findings are not intended to establish causal relationships but rather to provide in-depth insights that can inform future research.

Although the findings were largely consistent with the theoretical framework, it is important to note that the interview guide primarily focused on motivational experiences related to the adoption and maintenance of HLB. The questions were not specifically designed to explore failed attempts or persistent difficulties in maintaining these behaviours. As a result, contradictory or negative cases may be underrepresented in the data. Future research could benefit from explicitly examining barriers, lapses, and challenges associated with maintaining HL in order to provide a more comprehensive understanding of motivational processes.

Despite these limitations, the study offers valuable contributions for public health practice. The insights generated may support the design of culturally sensitive, autonomy-supportive, and motivation-enhancing interventions, particularly in contexts where sustained behaviour change is essential for reducing the burden of chronic disease.

Another limitation concerns the distribution of topics discussed during the interviews. Participants tended to speak more extensively about physical activity than about diet or nutrition. Although nutrition was addressed, it was less prominent in participants’ narratives. This may reflect both the focus of their lived experiences and the emphasis of the interview prompts. Future research could explore dietary practices in greater depth to provide a more balanced understanding of HLB.

### Implications of the findings

The findings of this study have several important implications for public health practice. They indicate that the sustainability of HLB is closely related to the quality of motivation rather than the quantity of motivation alone. Supporting individuals’ basic psychological needs for autonomy, competence, and relatedness is therefore essential for designing effective public health programs.

First, integrating autonomy-supportive approaches into primary care and community health services may strengthen individuals’ internalization of HLB. Counselling practices that acknowledge individuals’ personal values and preferences are more likely to promote long-term adherence to healthy behaviours. Second, social support emerged as a powerful motivational resource. Community-based initiatives that encourage group participation, family involvement, and peer support can play an important role in sustaining HLB.

Third, the finding that perceived health risks and illness experiences often act as turning points indicates that clear, ethical, and evidence-based risk communication strategies are critical in public health campaigns. Fourth, the influence of cultural and spiritual values suggests that culturally sensitive health communication may enhance the effectiveness of interventions in societies where such beliefs are strongly embedded. Finally, the motivational role of digital media highlights the potential of digital.

## Conclusion

This study explored the motivational processes underlying the adoption and maintenance of HLB within the framework of SDT. The results indicate that motivation for HL is shaped by a complex interaction of intrinsic and extrinsic factors that evolve through internalization over time. Satisfaction of the needs for autonomy, competence, and relatedness emerged as a central factor supporting sustained engagement in health-promoting behaviours.

External influences, such as social approval, professional guidance, cultural and spiritual beliefs, and digital media, were found to initiate behaviour change. However, as individuals experienced the physical and psychological benefits of HL, these influences often became internalised and transformed into more autonomous forms of motivation. Furthermore, experiences of illness and perceived health risks were identified as important triggers for behaviour change, underscoring the importance of well-designed risk communication strategies in public health practice.

Overall, the findings demonstrate that motivation for HL is not solely an individual matter but is also shaped by social, cultural, and structural factors. Sustainable behaviour change therefore requires supportive environments and health systems that foster autonomy, competence, and social connection. Public health strategies that incorporate these motivational principles have the potential to achieve lasting impact and contribute to reducing the burden of chronic disease while improving population health.

## Supplementary Information

Supplementary Material 1.

## Data Availability

All data generated during this study are included in this published article.

## Abbreviations

HL: Healthy Living
HLB: Healthy Living Behaviours
SDT: Self-Determination Theory
