# Supplementary material for: Motivational dynamics of healthy living: a qualitative study based on self-determination theory
Source: BMC Public Health. 2026 Apr 29;26:1863. doi: 10.1186/s12889-026-27586-9 (PMC13267382; doi:10.1186/s12889-026-27586-9)
Supplement: Supplementary file 1 — Supplementary Material 1. [file 12889_2026_27586_MOESM1_ESM.docx]

**Interview Questions**

Age:
Gender:
How long have you been adopting healthy lifestyle behaviors?
How many days per week do you engage in physical activity?
How many meals do you consume per day?
Do you consume snacks between meals?

**1. What factors motivate you to live a healthy lifestyle?**

**Follow-up Question 1:** What motivates you to engage in physical activity?
**Follow-up Question 2:** What motivates you to eat healthily?
**Follow-up Question 3:** What motivates you to be present in healthy environments (social media/TV/social settings)?

**2. Could you describe your process of developing healthy lifestyle behaviors?**

**Follow-up Question 1:** Could you describe the events that led you to make behavior changes?
**Follow-up Question 2:** Could you describe the people who encouraged you to adopt a healthy lifestyle?

**3. Could you describe how a healthy lifestyle has been reflected in your life?**

**Follow-up Question 1:** What positive or negative changes has a healthy lifestyle brought to your life?
**Follow-up Question 2:** How has a healthy lifestyle influenced your interpersonal relationships?
**Follow-up Question 3:** How has a healthy lifestyle affected your stress level?
**Follow-up Question 4:** How has a healthy lifestyle affected your sleep patterns?
